# Forward treatment planning techniques to reduce the normalization effect in Gamma Knife radiosurgery

**DOI:** 10.1002/acm2.12193

**Published:** 2017-09-27

**Authors:** Hao‐Wen Cheng, Wei‐Lun Lo, Chun‐Yuan Kuo, Yu‐Kai Su, Jo‐Ting Tsai, Jia‐Wei Lin, Yu‐Jen Wang, David Hung‐Chi Pan

**Affiliations:** ^1^ Department of Radiation Oncology Shuang Ho Hospital Taipei Medical University Taipei Taiwan; ^2^ Department of Neurosurgery Shuang Ho Hospital Taipei Medical University Taipei Taiwan; ^3^ Gamma Knife Center Shuang Ho Hospital Taipei Medical University Taipei Taiwan; ^4^ Department of Medical Imaging and Radiological Technology Yuanpei University of Medical Technology Hsinchu Taiwan; ^5^ Department of Surgery School of Medicine College of Medicine Taipei Medical University Taipei Taiwan

**Keywords:** Gamma Knife, normalization effect, stereotactic radiosurgery, treatment planning

## Abstract

In Gamma Knife forward treatment planning, normalization effect may be observed when multiple shots are used for treating large lesions. This effect can reduce the proportion of coverage of high‐value isodose lines within targets. The aim of this study was to evaluate the performance of forward treatment planning techniques using the Leksell Gamma Knife for the normalization effect reduction. We adjusted the shot positions and weightings to optimize the dose distribution and reduce the overlap of high‐value isodose lines from each shot, thereby mitigating the normalization effect during treatment planning. The new collimation system, Leksell Gamma Knife Perfexion, which contains eight movable sectors, provides an additional means to reduce the normalization effect by using composite shots. We propose different techniques in forward treatment planning that can reduce the normalization effect. Reducing the normalization effect increases the coverage proportion of higher isodose lines within targets, making the high‐dose region within targets more uniform and increasing the mean dose to targets. Because of the increase in the mean dose to the target after reducing the normalization effect, we can set the prescribed marginal dose at a higher isodose level and reduce the maximum dose, thereby lowering the risk of complications.

## 1 INTRODUCTION

Gamma Knife radiosurgery (GKRS) was initially used to treat deep intracranial lesions of limited volume. Over the last decade, an increasing number of studies have shown that large intracranial lesions with a volume of 20–30 cm^3^ can also be treated using GKRS.^1^, ^2^, ^3^, ^4^ Typically, multiple shots are required to cover target volumes (TVs) for treating large targets. Moreover, multiple shots are also used for targets with irregular contours to achieve improved target conformity. However, large or irregularly shaped targets are at an increased risk of radiation‐induced complications because more normal tissues inside or surrounding the targets are irradiated.^1^, ^2^, ^3^, ^4^ Ideally, the maximum treatment dose should be decreased to reduce the risk of complications, while concurrently maintaining a sufficiently high target dose.^1^, ^5^ In clinical practice, the main challenge is that multiple shots reduce the coverage of high‐value isodose lines (e.g., 90% and 70%) within targets; therefore, maintaining a sufficient target dose with a lower maximum dose is difficult.

**Figure 1:**
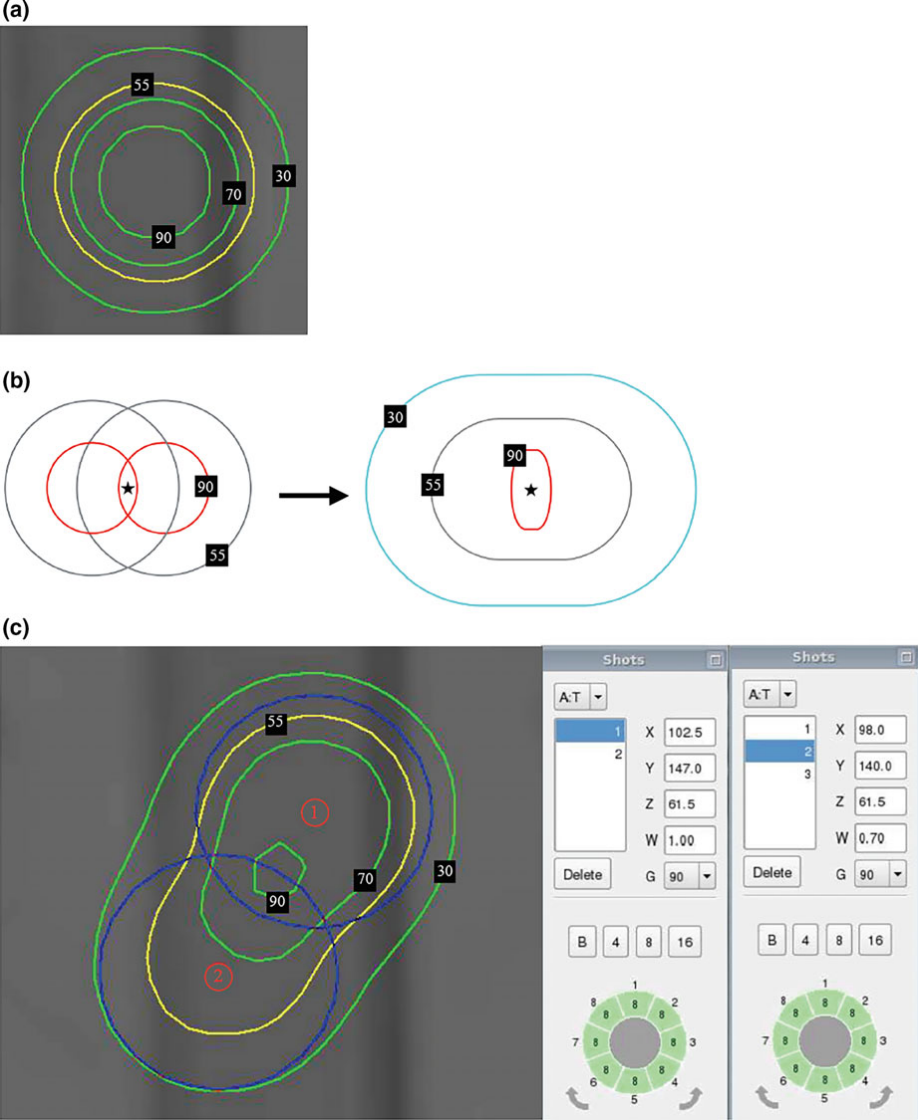
(a) A single shot has the largest proportion of high‐value isodose line coverage. (b) Normalization effect. Two shots are placed close to each other, and the sum of their contributions was considered; star sign represents the maximum dose point contributed by the shots. (c) Normalization effect caused by the interaction of two shots in the treatment plan. Note that the 90% and 70% isodose lines are smaller after the normalization effect of the two shots.

In forward treatment planning, a single shot of radiation delivers the most concentrated dose to the target, as depicted in Fig. 1(a). A single‐shot radiation pattern is characterized by its large portion of higher isodose lines in a small treatment volume, which permits uniform high‐dose radiation to the target with a steep dose gradient and sharp fall‐off of the dose outside the target margin.^6^ On the basis of this concept, for situations where multiple shots must be used for the treatment of large or irregularly shaped targets, creating a radiation field that is generated by multiple shots but mimics the dose distribution of a single shot has been suggested. The dose distribution should consist of a large portion of higher isodose lines, which can conform to the target shape during treatment planning.

Achieving conformal coverage of the target with a large portion of higher isodose lines under the prescribed dose (PD) would enable the target to receive a more homogeneous high‐dose radiation with an increased mean dose. When the target receives an increased mean dose, we can optimize the treatment dose by reducing the maximum dose delivered to the center of the target while maintaining the same dose in the margin. Accordingly, the surrounding normal tissues would receive less radiation, which would reduce the risk of complications.

While using multiple shots during treatment planning, however, a decrease in the proportion of the higher isodose coverage on the target should be avoided. In clinical practice, we generally define this phenomenon as the “normalization effect” between shots, as depicted in Fig. 1(c). Jitprapaikulsarn^7^ described the normalization effect as the formation of a “hot spot” influenced by the locations and magnitude of the maximum doses of shots. In this report, we use a clinical definition to describe the normalization effect. The normalization effect occurs because the isodose lines from each shot overlap and dose contributions between shots are added.^7^ After the interaction of two shots, the maximum dose (100% isodose level) in the new radiation field is renormalized and the shapes of the isodose lines change. Figure 1(b) illustrates the process of the normalization effect. Apparently, the normalization effect reduces the coverage proportion of 90% and 70% isodose lines.

The new collimation system of the Leksell Gamma Knife (LGK) Perfexion (PFX), consists of eight movable sectors, in which the collimator size can be adjusted among four settings (4, 8,16 mm, and blocked) independently and automatically. The PFX collimation system facilitates not only an increase in treatment efficiency but also the generation of composite shots to achieve more conformal treatments.^8^, ^9^

Studies on normalization effect reduction are scant. Therefore, this study evaluated the performance of forward treatment planning with and without the PFX collimation system for the normalization effect reduction. In addition, the clinical significance of the decreased normalization effect during treatment planning was elucidated.

## 2 MATERIAL AND METHODS

**Figure 2:**
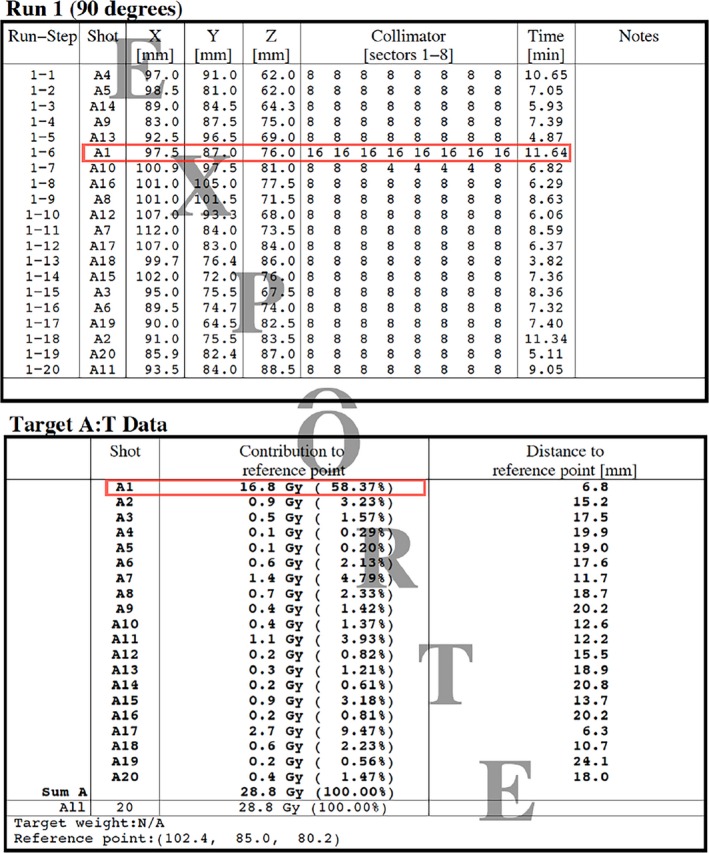
Example of a Gamma Knife treatment plan for a target with a volume of 10.2 cm^3^, using one large shot with a 16‐mm collimator, eighteen 8‐mm small shots, and one composite shot with 8‐mm and 4‐mm collimators. A1 is the main shot of this treatment plan. We maintained the maximum contribution of A1 to the reference point during treatment planning.

Our Gamma Knife center is equipped with a PFX and GammaPlan 9.0 treatment planning system. In forward treatment planning using multiple shots for radiosurgery, we typically first place a main shot in the region of the target center and choose a best‐fit collimator size for the main shot to cover the TV in the most thorough and conformal manner possible. Subsequently, we cover the remaining TV by adding multiple small shots to generate an ideal isodose line to fit the shape of the target margin. In treatment planning, the contribution of the radiation dose from the main shot is generally the maximum in the dose distribution. Figure 2(a) depicts an example of a dose plan with a 16‐mm large shot (A1), which delivers the maximum contribution to the reference point. For some large targets, we may use several large shots that deliver the greatest contribution to the reference point.

During treatment planning, frequent monitoring should be performed to detect the occurrence of the normalization effect after the delivery of a new shot that causes the shrinkage of the higher isodose lines covering the target. To minimize the normalization effect of the dose distribution, we intended to reduce the overlap of the higher isodose lines between shots. During multiple‐shot treatment planning, we adjusted the position and weighting of each shot to allow the 50–60% isodose lines to fit the target margin and the 70% isodose line to cover approximately 70–80% of the TV.^10^, ^11^ For the adjustment of shot positions, our strategy was to separate each shot appropriately to reduce the overlaps of higher isodose lines. For the adjustment of shot weightings, our strategy involved reducing certain shot weightings for attaining a lower dose contribution and less volume coverage to reduce the overlap of higher isodose lines.

After the normalization effect reduction, we can increase the proportion of coverage of high‐value isodose lines on the target. Once we achieve this goal which means that the uniformity of the high‐dose region within the target would increase and the mean dose to the target would increase; then we can set the PD at a higher isodose level with an acceptable target coverage (at least 95% of the TV covered by the PD in our treatment plans). Because we set out prescription marginal dose at a higher isodose level, the maximum dose can be reduced.

The PFX collimation system provides another method for reducing the normalization effect, which entails using composite shots. Our strategy involved the selection of some sectors with smaller collimator sizes (4 or 8 mm) or a blocked collimator in the junction of the large shots. This concept of combining sectors to form a composite shot is similar to the adjustment of shot weightings mentioned previously, but reduces the dose in a shot only partially from parts of sectors with smaller collimators.

**Figure 3:**
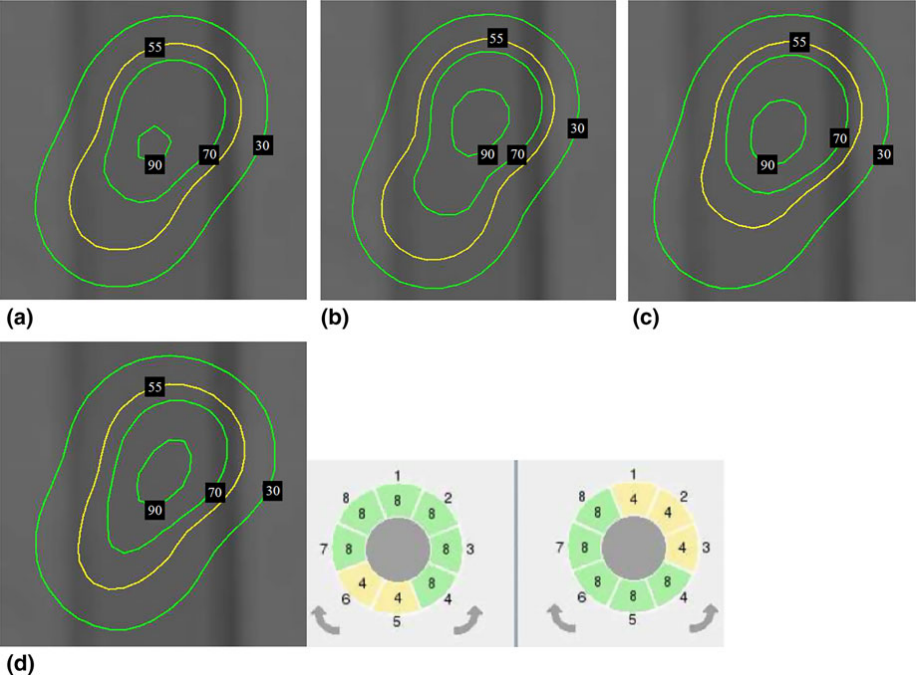
(a) Radiation field contributed by two shots before normalization effect reduction. The normalization effect caused by two shots can be reduced by adjusting shot positions or weightings or using composite shots. (b) For the adjustment of shot positions, shot 2 was adjusted in the y‐direction 1 mm posterior to shot 1. (c) For the adjustment of shot weightings, we adjusted the weighting of shot 2 from 0.7 to 0.5. (d) For using composite shots, we selected some sectors in the junction of these two shots with smaller collimator sizes (4 mm).

During dose planning, if shrinkage of higher isodose lines coverage occurs on the target, we should determine which shot other than the main shot has a greater contribution to the reference point compared to that from other shots. If a small, non‐main shot is excessively strong such that it causes the normalization effect in the dose distribution, we should adjust the position of that shot away from the main shot, reduce its weighting, or use composite shots. Figure 3 shows the different forward treatment planning methods for the normalization effect reduction.

## 3 RESULTS

**Figure 4:**
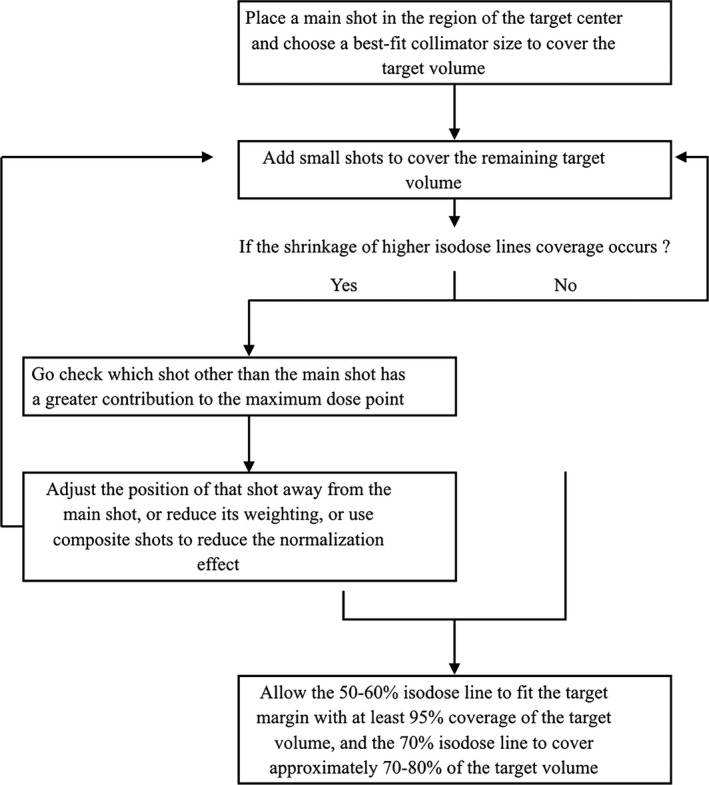
A flow chart illustrating the principal steps of our treatment planning to reduce the normalization effect in GKRS.

Figure 4 shows our principal treatment planning steps to reduce the normalization effect in GKRS.

We introduced two case examples [acoustic neuroma and arteriovenous malformation (AVM)] to demonstrate our treatment strategy in certain benign intracranial lesions with large treatment volumes. Table 1 presents the dosimetric comparisons between the treatment plans with and without the normalization effect reduction for these two cases.

**Table 1:** Dosimetric variables and treatment data in GKRS plans for an acoustic neuroma and an AVM

	Case (a) Acoustic neuroma		Case (b) AVM	
	Plan (1)	Plan (2)	Plan (3)	Plan (4)
TV (cm^3^)	11.5		26.9	
Prescribed dose (Gy)	11.5		16	
Prescribed isodose line (%)	58	51	55	51
Coverage (%)	96	96	96	96
Beam‐on time (min) (dose rate: 1.735 Gy/min)	110.8	90.6	246.6	206.5
Number of shots	22	19	29	26
Mean target dose (Gy)	15.6	15.3	22.2	21.2
Maximum dose (Gy)	19.8	22.5	29.1	31.4
PIV (cm^3^)	12.6	14.2	37.9	40
PIV_50%PD_ (cm^3^)	33.4	39.7	116.4	129.3
GI = PIV_50%PD_/PIV	2.65	2.8	3.07	3.23
CI = (TV_PIV_/TV) × (TV_PIV_/PIV)	0.84	0.74	0.66	0.63
The percentage of TV covered by the 70% isodose line (%)	74	39	76.3	40
The volume of TV (cm^3^) covered by 15 Gy (130% of the PD) in case (a), and 21 Gy (130% of the PD) in case (b)	7.0	6.1	17.6	14.4
	The volume of brainstem receiving 50% of the PD (cm^3^)		12‐Gy volume (cm^3^)	
	2.9	3.5	60.9	66.7

TV, target volume; PIV, prescription isodose volume; TV_PIV_, TV covered by the PIV; Coverage (%), (TV_PIV_/TV) × 100%; CI, conformity index; GI, gradient index; PD, prescribed dose; PIV_50%PD_, PIV covered by 50% of the PD.

### 3.A Case illustration

**Figure 5:**
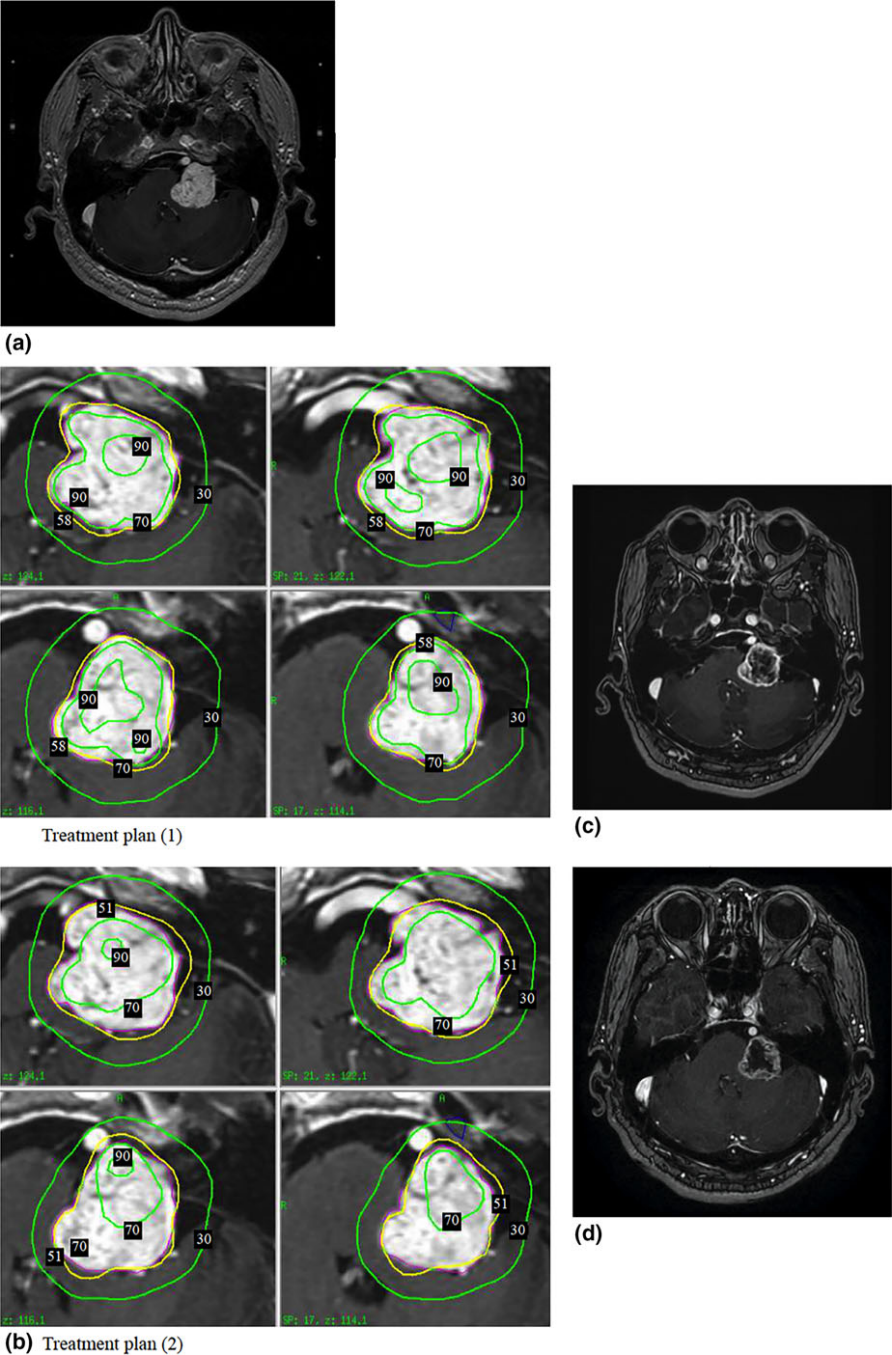
(a) A left acoustic neuroma with a volume of 11.5 cm^3^ was treated by GKRS. (b) Comparison of two treatment plans for this tumor, plan (1) with the normalization effect reduction and plan (2) without the normalization effect reduction. The tumor was treated by using treatment plan (1). (c) Follow‐up MRI of the same tumor at 6 months after treatment revealed an obvious loss of contrast enhancement with shrinkage of the tumor volume from 11.5 to 8.7 cm^3^. No adverse radiation reactions were observed. (d) Follow‐up MRI 12 months after treatment showed that the tumor volume further regressed to 8.3 cm^3^.

**Case (a)**: A 60‐year‐old man with a large acoustic neuroma (tumor volume, 11.5 cm^3^) received GKRS as the primary treatment. Figure 5(a) shows the pre‐radiosurgical magnetic resonance imaging (MRI) result. Figure 5(b) depicts two treatment plans for comparison: treatment plan (1) with and (2) without the normalization effect reduction. Plan (1) was used as the treatment and included one 16‐mm shot, eleven 8‐mm small shots, and ten small composite shots with 8‐ and 4‐mm collimators. Plan (2) was only used for the test and included four 16‐mm and fifteen 8‐mm shots. Both targets (Fig. 5) were sufficiently covered by the prescribed isodose line; in plan (1) and (2), 96% of the tumor volume was covered by 58% and 51% isodose line, respectively. In both plans, the target margins received 11.5 Gy as the PD. Although the mean target doses of both plans were comparable (15.6 vs. 15.3 Gy), the maximum dose in plan (1) was smaller than plan (2) (19.8 vs. 22.5 Gy). After the treatment, the patient was followed up every 6 months. The 6‐month follow‐up MRI showed the tumor volume decreased from 11.5 to 8.7 cm^3^ with an obvious loss of contrast enhancement in the tumor [Fig. 5(c)], which is usually a favorable sign for long‐term tumor control.^3^, ^12^ The 12‐month follow‐up MRI [Fig. 5(d)] showed further tumor regression without side effects of swelling or perifocal edema.

**Figure 6:**
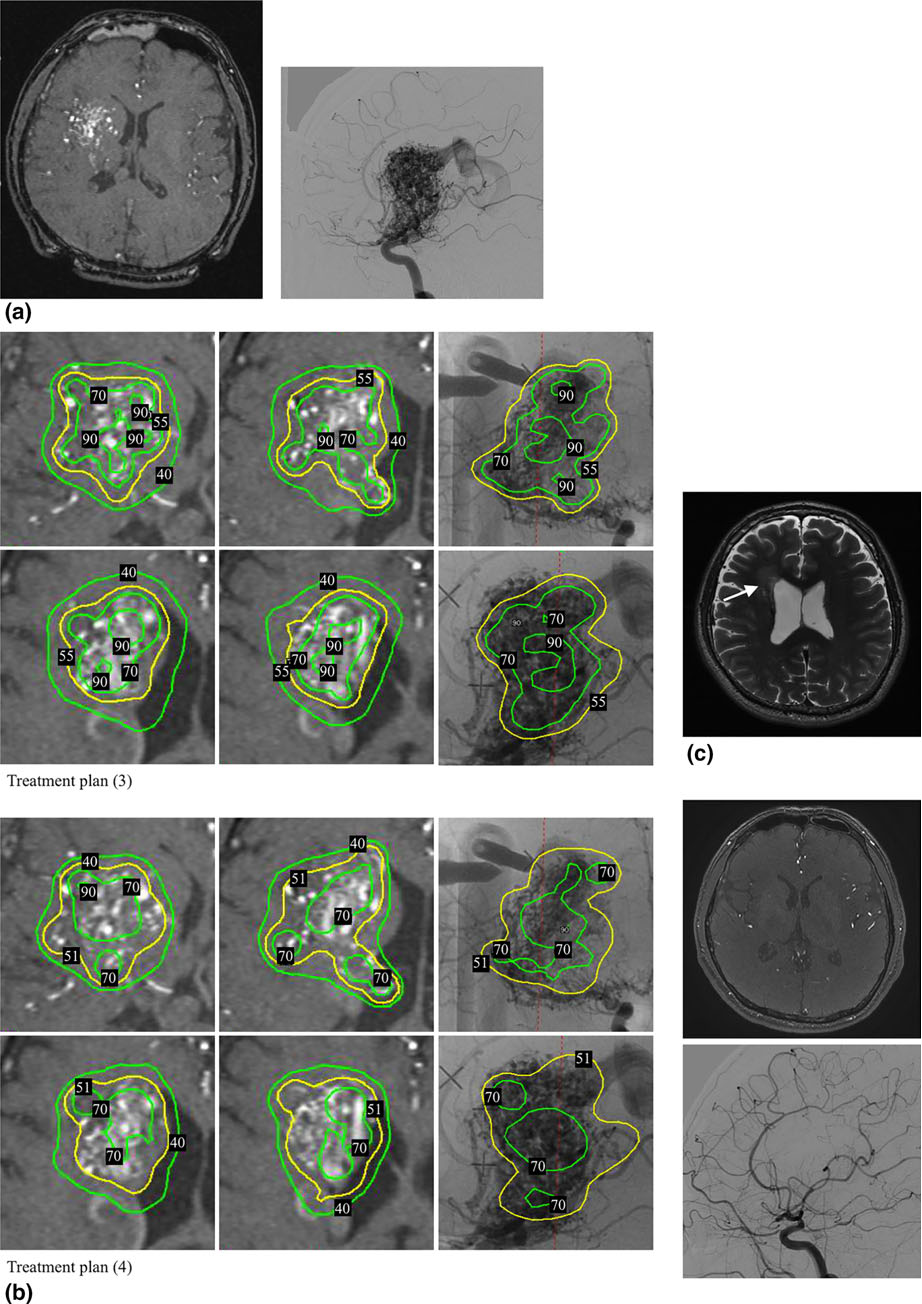
(a) An AVM with a volume of 26.9 cm^3^ was treated by GKRS. (b) Comparison of two treatment plans for this AVM, plan (3) with the normalization effect reduction and plan (4) without the normalization effect reduction. The AVM was treated by using plan (3). (c) Follow‐up T2 MRI 12 months after treatment revealed a partial regression of the AVM nidus with temporary and mild radiation‐induced edema (white arrow), but had no neurological symptoms. (d) Follow‐up TOF MRI and cerebral angiogram 19 months after treatment revealed a complete obliteration of the AVM.

**Case (b)**: A 32‐year‐old man with an AVM (estimated nidus volume, 26.9 cm^3^) in the right temporal lobe and basal ganglia received GKRS as the primary treatment. The pre‐radiosurgical MRI result and cerebral angiogram are shown in Fig. 6(a). Figure 6(b) shows the comparison of the two treatment plans, denoted as (3) with and (4) without the normalization effect reduction. Both targets were sufficiently covered by the prescribed isodose lines; in plan (3) and (4), 96% of the TV was covered by 55% and 51% isodose line, respectively. Plan (3) consisted of four large shots (two 16‐mm shots and 2 composite shots with 16‐ and 8‐mm collimators) and twenty‐five 8‐mm small shots. Plan (4) included eight large shots (seven 16‐mm and one composite shot with 16‐ and 8‐mm collimators) and eighteen 8‐mm small shots. The AVM was treated with a PD of 16 Gy at the 55% isodose line by using plan (3). The 12‐month follow‐up T2 MRI [Fig. 6(c)] revealed an apparent partial regression of the AVM, only with mild radiation‐induced edema and without any clinical neurological symptoms. The 19‐month follow‐up time‐of‐flight (TOF) MRI and cerebral angiogram showed a complete obliteration of the AVM.

## 4 DISCUSSION

According to the literature, radiosurgical treatments for large‐volume tumors or AVMs may sustain a higher risk of complications, such as tumor swelling or brain edema.^1^, ^2^, ^3^, ^10^, ^13^ Ideally, a conformal and sufficient target dose should be maintained for effective treatment. However, the maximum dose should be reduced to avoid the risk of complications. In GKRS, the normalization effect reduction during treatment planning can help achieve the aforementioned dose distribution.

In this report, we proposed different methods of forward treatment planning that can reduce the normalization effect. We may adjust shot positions and weightings to prevent the normalization effect of the dose distribution in models of LGK without the new collimation system. The PFX collimation system not only facilitates the achievement of a more conformal treatment to the target but also provides an alternative method for reducing the normalization effect by using composite shots. The reduction of the normalization effect can increase the proportion of coverage of higher isodose lines on the target. Therefore, if we set the same marginal dose to the target, the uniformity of the high‐dose region within the target would increase and the mean dose to the target would increase. As a result of such homogeneous high‐dose, and conformal irradiation to the target, a PD can be set at a higher isodose level with an acceptable target coverage (at least 95% of the TV covered by the PD in our treatment plans). Therefore, the maximum dose delivered to the target center can be reduced, and a sufficient mean target dose can be maintained. Theoretically, because the maximum dose is reduced, the surrounding normal tissues receive low radiation, thus lowering the risk of complications.

Conventionally, the PD to the target margin is set at the 50% isodose level because of the rapid fall‐off of the dose outside the target margin.^14^, ^15^ From our experience, we suggest that a large‐volume target should be treated with the PD at a slightly higher isodose level (55–60% in most cases); moreover, the ideal target conformity and sharp dose gradient are maintained. This strategy can reduce the maximum dose and prevent an overdose to the target.

In this report, we presented two case examples of large‐volume lesions treated with GKRS. The dosimetric variables of the treatment were compared between the two treatment plans (with and without the normalization effect reduction; Table 1). The variables included the conformity index (CI; based on the Paddick CI),^16^ gradient index (GI; based on the Paddick GI),^14^ mean target dose, maximum dose, prescription isodose volume (PIV), PIV covered by 50% of the PD (PIV_50%PD_), percentage of TV covered by the 70% isodose line, the volume of TV covered by 15 Gy (130% of the PD) in case (a) and 21 Gy (130% of the PD) in case (b), the volume of brainstem receiving 50% of the PD, and 12‐Gy volume that correlates with the risk of radiation necrosis.^17^, ^18^

**Case (a)**: In both treatment plans, the target margins received 11.5 Gy. Although plan (1) had a higher isodose level at the target margins than plan (2) (11.5 Gy at 58% vs. 11.5 Gy at 51%), plans (1) and (2) had comparable mean target doses (15.6 vs. 15.3 Gy), and plan (1) had a lower maximum dose than plan (2) (19.8 vs. 22.5 Gy). Plan (1) had a more uniform 70% isodose line coverage (74% vs. 39% of the TV) with a larger volume of the TV covered by 15 Gy (130% of the PD) than plan (2) did (7.0 vs. 6.1 cm^3^), and a larger 90% isodose line coverage. Furthermore, plan (1) had a slightly better CI (0.84 vs. 0.74) and GI (2.65 vs. 2.8) than plan (2) did. With respect to critical organ sparing, plan (1) with a lower maximum dose had a smaller volume of brainstem receiving 50% of the PD (2.9 vs. 3.5 cm^3^).

**Case (b)**: Plan (3) had a higher mean target dose (22.2 vs. 21.2 Gy) with a lower maximum dose (29.1 vs. 31.4 Gy) than plan (4). After the normalization effect reduction, plan (3) had a more uniform 70% isodose line coverage (76.3% vs. 40% of the TV) and a larger volume of the TV covered by 21 Gy (130% of the PD) than plan (4) did (17.6 vs. 14.4 cm^3^). Plan (3) also revealed a more favored CI (0.66 vs. 0.63) and GI (3.07 vs. 3.23) than plan (4) did. Both plans had the same PD (16 Gy). However, because the maximum dose in plan (3) was lower than plan (4) due to a higher isodose level at the target margins (55% vs. 51%), the 12‐Gy volume affecting the brain tissues was smaller in plan (3) than in plan (4) (60.9 vs. 66.7 cm^3^).

During treatment planning, using techniques to reduce the normalization effect can improve target coverage at higher isodose levels. However, some possible drawbacks are concerned. For the adjustment of shot positions, the radiation volume may increase and surrounding normal tissue may receive an excessive dose of radiation. If there are critical structures in the vicinity, radiation dose limits for these structures must not be exceeded.

Reducing certain shot weightings during treatment planning can cause a reduction in the target coverage due to less isodose line coverage. This would result in the need for additional shots to achieve target coverage. When more shots are used, the normal tissue may receive a higher dose because of scattering and leakage of radiation while patients are repositioned between shots.^19^, ^20^ Moreover, the beam‐on time may increase with an increasing number of shots.

The use of composite shots by arranging smaller collimators in the junction of two large shots may prevent the normalization effect. However, the beam‐on time must be increased due to the lower output of the smaller collimators used for composite shots. In our two illustrated cases, a slightly higher treatment time [case (a), 110.8 vs. 90.6 min; case (b), 246.6 vs. 206.5 min] and more shots [case (a), 22 vs. 19; case (b) 29 vs. 26] were required for the treatment plans with the normalization effect reduction than for the treatment plans without the normalization effect reduction. However, both cases tolerated the treatment procedure well and obtained desirable clinical outcomes.

## 5 CONCLUSIONS

We proposed different forward treatment planning techniques to reduce the normalization effect during forward treatment planning by adjusting shot positions and shot weightings and by using composite shots. The reduction of the normalization effect increases the proportion of coverage of higher isodose lines on the target; thus, the mean dose to the target increases. This increased homogeneous radiation maintains a sharp dose gradient and conformal treatment to the target. Through this method, we can maintain a sufficient mean treatment dose for a large or irregularly shaped tumors or AVM. Moreover, the maximum dose of the treatment can be reduced by setting an effective marginal dose at a higher isodose level; while normal tissues receive less radiation, the risk of complications may be lowered.

## CONFLICT OF INTEREST

The authors declare that they have no conflicts of interest.
